# Physics, chemistry and biology of functional nanostructures II

**DOI:** 10.3762/bjnano.5.134

**Published:** 2014-08-06

**Authors:** Anatolie S Sidorenko

**Affiliations:** 1 Ghitu Institute of Electronic Engineering and Nanotechnologies of ASM, Chisinau, Moldova

**Keywords:** nanotechnologies, nanomaterials, sensors

Nanotechnology gives to the 21^st^ century the “NANO” logo and covers all domains of human activity ranging from electronics and medicine, to aerospace and agriculture. One of the creators of large-scale nanotechnological implementations Mihail Roco, the initiator of the National Nanotechnology Initiative, a United States federal government program, mentioned that “The accelerating pace of discovery and innovation and its increasingly interdisciplinary nature leads, at times, to the emergence of converging areas of knowledge, capability and investment; nanotechnology is a prime example. It arose from the confluence of discoveries in physics, chemistry, biology and engineering.” [1]. Starting in 2000 as the National Nanotechnology R&D Programs, first in the United States, then in Germany, and now in over 60 countries all over the world, nanoscience and nanotechnology gathered impetus and is one of the most rapid developing areas today. The intrinsic logic of that development determines the route of the progress: from a simple, evolutionary reduction of the structure size of single elements (for example, the size of the elementary transistor in a microchip) toward the revolutionary introduction of self-assembling nanostructures and functional nanomaterials.

The self-organization of nanoparticles and nanotubes and the introduction of those in various materials allowed for solutions to problems which have been present for a long time. An example is the problematical increase of the critical current in new MgB_2_ superconducting material. This superconductor is very promising for technical applications due to its high critical current of up to 10^7^ A/cm^2^. However, this critical current is only present in the MgB_2_ superconducting material when there is no magnetic field. The external magnetic field very rapidly suppresses the critical current and destroys the superconductivity of magnesium diboride. This issue was successfully resolved by the introduction of self-assembled magnetic nanodots on the MgB_2_ surface, which gives rise to a new hybrid material with the desired properties [2].

The conceptual idea of this Thematic Series saw the light of day at the international conference bearing the “NANO” logo, which took place in September 2013 in Chisinau, Moldova (NANO-2013). A variety of technological approaches for the assembly of functional nanostructures and nanomaterials with tailored properties was presented at this conference.

We would like to thank all colleagues who developed new ideas, novel approaches and devices, and submitted their valuable contributions to this Thematic Series. I am convinced that this Thematic Series will attract the attention of researchers and will find many readers! The professional and dependable editorial support by the Editorial Office of the Beilstein-Institut is greatly acknowledged.

Anatolie S. Sidorenko

Chisinau, June 2014
